# The beating heart of melanomas: a minor subset of cancer cells sustains tumor growth

**DOI:** 10.18632/oncotarget.259

**Published:** 2011-04-10

**Authors:** Patrick Schmidt, Hinrich Abken

**Affiliations:** Tumor Genetics, Department I of Internal Medicine and Center for Molecular Medicine Cologne, University of Cologne, D-50931 Cologne, Germany

**Keywords:** melanoma, cancer stem cell, adoptive cell therapy

## Abstract

The recent observation that targeted elimination of a minor subpopulation of melanoma cells can lastingly eradicate the tumor lesion provides strong evidence that an established melanoma lesion is hierarchically organized and maintained by definite subset of cells but not by every random cancer cell. This review discusses the concepts of discrete cancer stem cells and of a cellular hierarchy in melanomas, the rationale for shifting therapies from broad tumor cell cytotoxicity into selective cancer cell elimination strategies and the challenges for future therapeutic concepts.

## INTRODUCTION

Current regimens in cancer therapy attempt to eradicate all the malignant cells in a tumor lesion; this is based on the assumption that each cancer cell has equal malignant capacities. However, it has long been known that tumor lesions display an enormous histological heterogeneity; the tumor cell mass of most malignant lesions consists of a variety of cancer cells with different proliferative capacities, some of which are still present in a postmitotic stage. Genetic differences and increasing genetic instabilities are thought to drive such phenotypic heterogeneity; this results in a variety of different cell clones, which populate the tumor cell mass. The accumulation of oncogenic and tumor repressor gene mutations in an increasing number of cancer cells during tumor progression points toward a multi-hit process which drives the clonal evolution of malignant cell clones by stepwise acquisition of mutations, as formulated by Fearon and Vogelstein (1990) [1] for colon cancer. An overwhelming body of evidence, which was collected in the following years, strongly supports the clonal evolution model in tumor progression. Recent advances in global genome sequencing confirmed the presence of genetic heterogeneity in primary tumors and identified driver mutations in metastasis in addition to common mutations [2].

A variable but low number of cells isolated from solid tumor lesions can initiate tumors of the same histological heterogeneity as the parental tumor. Data based on xeno-transplantation combined with clinical observations fueled the cancer stem cell (CSC) model with the central paradigm that tumor initiation and progression is driven by a minor subset of discrete CSCs which have the capacity to renew themselves and to establish tumors upon transplantation, to stay quiescent (“dormant”) for long time, to be more resistant to chemotherapy and radiation, and to drift to distant sites of the body to initiate metastases. In this context, the CSC paradigm describes several phenomena in tumor biology which have been clinically observed but are poorly understood. For instance, metastatic relapse of melanoma can occur more than a decade after curative surgical treatment of the primary lesion; this phenomenon is thought to be due to the same cancer-originating cell, which drives cancer progression and relapse [3].

Recent data from our group [4] provide strong evidence that a minor subset of melanoma cells drives melanoma progression. Targeting CD20-positive melanoma cells, which constitute as little as 0.1 – 2% of the cancer cell population, can completely and lastingly eradicate the disease in mice without the need to target the tumor cell mass. In this review we discuss some consequences and challenges for the understanding of melanoma biology and for the future design of therapeutic regimes.

## EVIDENCE FOR THE CANCER STEM CELL PARADIGM

Observations by Pierce and co-workers that teratomas contain pluripotent stem cells led to the early definition of CSCs [5, 6]. The concept was subsequently sustained by deciphering the hierarchical organization in hematological malignancies. In their landmark paper Bonnet and Dick (1997) identified CSCs in most types of acute myeloid leukemia by xeno-transplantation and calculated their frequency to be approximately 10^−6^ [7, 8]. Subsequently, tumor-inducing cells were identified in solid tumors including the mammary carcinoma. Cells with CD44^+^ CD24^−/low^ phenotype induced tumors upon transplantation, whereas the majority of cancer cells from the same lesion did not [9]. The induced tumors displayed the same histology as the parental tumor and harbored tumorigenic cells, which again induced tumors upon serial transplantation. Since no clear morphological or phenotypical genotype can be attributed to them, CSCs are functionally identified by their ability to induce tumors upon transplantation into mice with different grades in immune deficiency by limiting dilution, most rigorously by transplantation of a single cell (for review [10, 11]). The induction of progressively growing tumors which recapitulates the parental tumor in its cellular heterogeneity reflects self-renewal of CSCs and their ability to differentiate. Long-term self-renewal has been confirmed by serial transplantation of re-isolated CSCs, which gives rise to secondary tumors. However, the conclusions drawn thereon are based on some premises which led to controversial conclusions in the definition of CSCs in general and in melanomas in particular [12-16].

One reason for the controversial discussion is the existence of major differences in the experimental context. At least the assay duration, the degree of immune deficiency, and the environment into which melanoma cells are transplanted are critical parameters, which substantially impact on the outcome of the assay. A recent study by Weissman and colleagues calculated a frequency of tumorigenic cells in any melanoma of about 1/2000 cells [17]. Although this study indicates that tumor-initiating potential is rare in melanomas, testing under modified conditions revealed that approximately 1 out of 6 (1/2 – 1/15) melanoma cells is capable of inducing tumors [18]. In line with that, a high incidence of tumor formation upon transplantation of single cells from BRAF mutated, PTEN- melanomas was reported [19].

The use of the transplantation assay to identify cancer stem cells has substantial limitations. First, human cells have to overcome species barriers upon transfer into the murine environment. At least for some solid cancers, mouse tumor cells accordingly showed improved engraftment when transplanted into fully compatible hosts [20-23], whereas this was not the case for murine leukemias [24-26]. Second, transplanted cells have to reconstruct their own niche, which will be different from the particular environment in an existing tumor from which they were isolated. As a consequence, the transplanted cell is more highly challenged for functional flexibility upon transplantation than the same cell resident in the tumor tissue. Therefore, the transplantation assay does not address whether or not an established melanoma is organized in a functional hierarchy with different cancer cell subsets. Not every cell exhibiting tumorigenic potential in the transplantation assay will contribute to melanoma progression. Once the tumor tissue is established, the tumor-initiating potential may be repressed by intrinsic cellular mechanisms due to clonal evolution processes or epigenetic changes; extrinsic mechanisms such as metabolic insufficiencies or low oxygen may additionally contribute to this. Recent data from our group support the assumption that, once established, melanoma maintenance depends on only a minority of melanoma cells. The latter has to be eliminated in order to eradicate an established tumor lesion [4].

**Figure 1:**
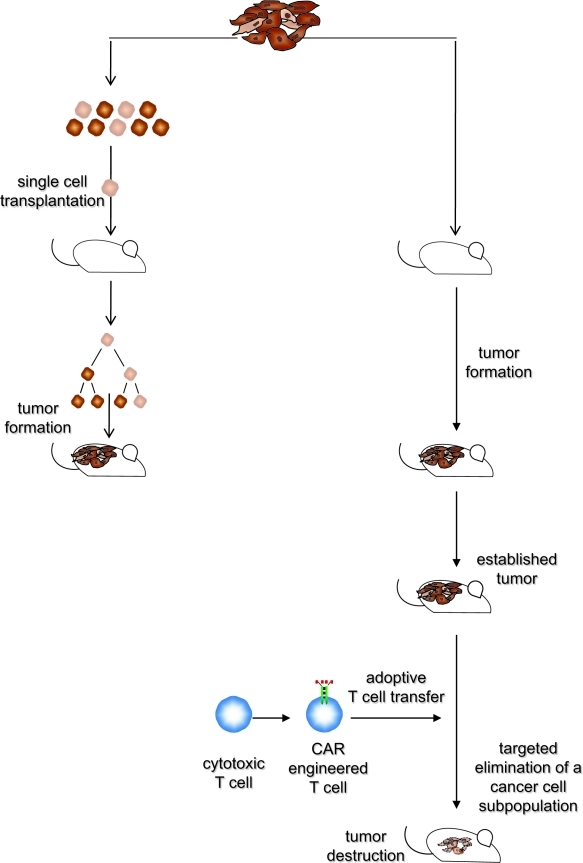
The hierarchical and the transplantation model may reflect different stages in melanoma development Transplantation assays indicate that isolated melanoma cells with different phenotypes can initiate by asymmetric cell divisions new tumor lesions when transplanted under appropriate conditions. This provides evidence for a variable, potentially high number of cancer cells with tumor-originating capabilities. Once established, targeted elimination of a definite, non-random minor subset of melanoma cells results in melanoma eradication, which provides evidence for a functional hierarchy with a “master cell” required for maintaining the melanoma lesion. Both models may reflect different stages in melanoma development. Although the mass of cancer cells in an established tumor lesion does not provide melanoma-maintaining functions, functional plasticity enables individual cells to exhibit melanoma-originating capabilities when isolated from the tumor tissue and seeded under limiting dilution conditions. Once the tumor tissue has become re-established, a few “master cells” take over to maintain tumor integrity and to fuel progression.

## A MINOR SUBSET OF MELANOMA CELLS SUSTAINS TUMOR PERSISTENCE AND GROWTH

To date no direct evidence is available to show whether maintenance and long-term progression of established solid tumors is driven by all cancer cells or by a subset of them. Using in vivo targeting of defined tumor cell subsets, we recently demonstrated that selective elimination of a definite, minor cancer cell subpopulation is particularly effective in eradicating established melanoma lesions irrespective of the tumor cell mass [4]. Human melanoma lesions which recapitulate the histological heterogeneity of the parental tumor were established in immunodeficient mice by means of transplantation of unsorted biopsy cells. Once the tumor was established, i.e. growing progressively and connected to the host vasculature, pre-defined cancer cell subsets in the tumor tissue were specifically targeted by adoptive transfer of cytotoxic T cells, which were redirected in an antigen-restricted manner by a chimeric antigen receptor (CAR). The CAR is composed of an extracellular, antibody-derived binding domain and an intracellular signaling domain derived from the T cell receptor complex [27]. CAR-engineered T cells are thus antibody-redirected and MHC-independently activated to target cells which express the cognate antigen on the cell surface. Engineering with a CAR specific for high-molecular weight, melanoma-associated antigen (HMW-MAA), also known as melanoma chondroitin sulfate proteoglycan (MCSP), redirects cytolytic T cells to selectively eliminate HMW-MAA^+^ cells; T cells with a melanotransferrin (mtf) specific CAR eliminate mtf^+^ cells. Targeted elimination of melanoma cells with mtf expression, which constitute nearly all malignant cells in a melanoma lesion, eradicates the tumor lesion in immune deficient mice. However, the same effect was induced by targeted eradication of HMW-MAA^+^ cells, which make up about 40% of the malignant cells in the lesion. It is noteworthy that Melanoma lesions, were also eradicated by targeted elimination of a 0.1 – 2% subset of cancer cells which co-express CD20 together with HMW-MAA; no relapse occurred in more than 36 weeks. The unexpected observation is that elimination of the minor population of CD20^+^ cancer cells is as effective in tumor eradication as targeting the entirety of the cancer cells. In this context, it is of substantial relevance that melanoma-sustaining cells were identified without isolation from tumor tissues and transplantation into a xeno-environment. The effect is specifically associated with the CD20^+^ melanoma cell subset since elimination of any random 10% cancer cells from the established tumor lesion is not effective. A caveat is that obviously some melanomas, in our cohort 1 out of 5 patients, do not harbor CD20^+^ melanoma cells. In those melanoma lesions the cancer cell subset may not exist or may lack CD20. However, CD20 itself does not seem to be causally involved in the induction of tumor-sustaining capacities since transgenic expression of CD20 in a random, CD20-negative melanoma cell population and subsequent targeted elimination of those cells from an established melanoma did not eradicate the tumor lesion. Taken together the data provide the first direct evidence that an established melanoma lesion is hierarchically organized, i.e. harboring a minor subset of cancer cells which is crucial in maintaining malignant tumor growth. However, this observation raises the following implications with substantial impact for the understanding of melanoma biology.

### 1. Do CD20+ melanoma cells exhibit capacities of cancer stem cells?

The observation that continuous melanoma growth requires the presence of a definite minor subset of cells implies that those cells are capable of initiating new melanoma lesions when transplanted into an immune-deficient host. Transplantation of enriched HMW-MAA^+^ CD20^+^ melanoma cells into the immunodeficient mouse confirmed their tumor-inducing capabilities. Induced melanomas exhibited the same histological morphology with low numbers of CD20^+^ cells as the parental tumor; this is conserved in serial transplantation. CD20^+^ melanoma cells thereby exhibit cancer stem cell capabilitis. Similarly, Herlyn's group previously reported a tumorigenic potential in isolated CD20^+^ melanoma cells which were grown in spheroids in vitro [28]. These cells express melanoma-associated markers chondroitin sulfate proteoglycan (CSPG), β3 integrin and MCAM as well as stem cell markers including CD133. These cells can differentiate under appropriate conditions in vitro into multiple cell lineages and exhibit self-renewal capacities upon serial transplantation in vivo recapitulating the cellular heterogeneity of the parental tumor. The data are insufficient to determine whether these cells are the only cells capable for renewal and tumor induction in melanoma; other subsets of cells with the same stem cell capability might possibly exist simultaneously in the same tumor lesion.

### 2. Do CD20+ melanoma cells represent a stable melanoma cell lineage or a transient phenotype associated with cancer-maintaining capabilities?

Markers, and combinations of markers, which are used to isolate CSCs are currently chosen with respect to their heterogeneous expression in tumor tissues and not due to insight into a functional hierarchy of stem cells, for instance, CD24 and CD44 in breast cancer [9] and pancreas carcinoma [29], CD90 on hepatocellular carcinoma [30], CD133 in colon, lung, brain tumors [31-35], and EpCam in colorectal and pancreatic cancer [29, 36]. Expression of CD271 [17], ABCB5 [37], CD133 [38], and CD20 [28] by melanoma CSCs has been reported. However, the value of these and other markers in identifying CSCs has been a matter of controversy since Quintana et al. (2010) [39] showed a remarkable phenotypical plasticity of those cells, i.e. sorted marker-positive and marker-negative cells are similarly able to reconstitute a tumor with the same pattern of marker expression as the parental tumor. In particular, tumors which were initiated either by isolated CD133^+^ or CD133^−^ melanoma cells displayed the same heterogenous pattern in CD133 expression as the parental tumor [16, 18]. Moreover, melanoma cells expressing the H3K4 demethylase JARID1B, which are more tumorigenic than cells lacking JARID1B, can produce cells which lack JARID1B; the latter, however, can also produce JARID1B^+^ cells [40]. Similar observations were reported by Sharma et al. (2010) [41], i.e. that individual cells can transiently acquire or loose drug resistance mediated by JARID1A. In this context, the expression of certain markers by melanoma inducing cells may not be heritably fixed but rather determined as a result of a number of parameters provided by the environment.

Similarly, the CD20^+^ phenotype of an individual melanoma cell in an established tumor lesion may not be stable. Evidence for this is provided by tumors induced by transplantation of isolated CD20^+^ melanoma cells. Such tumors display a heterogeneous phenotype with the majority of CD20^−^ and a small subset of CD20^+^ melanoma cells [4, 28]. The underlying mechanism may be phenotype-switching associated with asymmetric cell division. The ability to switch phenotypes implies that most cells in the tumor tissue will have the potential to adopt a stem cell-like phenotype, independently of whether cells exhibit a more differentiated postmitotic or a more proliferative phenotype at a given moment (for review [42]). Assuming that melanoma-maintaining cells display their phenotype and functional capabilities in a reversible fashion, eliminating cells in the functional stage of tumor maintenance would only be sufficient to eradicate the established melanoma lesion if reversion to the stage of maintenance does not occur frequently.

### 3. How frequent are cells which maintain tumor progression?

Based on the transplantation assay, the estimated frequency of CSCs is commonly believed to be rare. However, this may be greatly underestimated; this became obvious when one out of ten murine leukemia cells induced leukemia in compatible hosts [43] and one out of four melanoma cells induced melanomas [18]. While transplantation assays score for self-renewal and tumor re-formation in a heterologous host, melanoma-sustaining cells were identified by targeted elimination of defined subsets of cancer cells [4]. The frequency of targeted CD20^+^ melanoma cells in tumor tissues was about 2% or less, whereas approximately 10^4^ enriched HMW-MAA^+^ CD20^+^ cells were required to induce melanomas upon xeno-transplantation. However, CD20-negative melanoma and other cancer types may harbor cancer-maintaining cells in different frequencies. In concurrence with others [11], we assume that the calculated frequency of tumor inducing cells as estimated by the limiting dilution transplantation assay is less relevant in the context of melanoma-maintaining cells in an established tumor lesion.

### 4. Are CD20+ melanoma cells good targets for therapy?

The data strongly imply that CD20^+^ melanoma cells are appropriate targets for therapy. However, therapeutic strategies have to deal with the particular properties of those cells and of cancer stem cells in general. Depending on their position in a functional hierarchy, targeting cancer cell subsets will have different therapeutic effects. When targeting a small subset of cancer cells, therapy may initially be accompanied by a slow increase in tumor progression until a decrease in tumor cell mass becomes obvious. Mathematical modeling revealed that strategies which eliminate tumor repopulating cells will be more successful than increasing the death rate or decreasing the production of mature tumor cells; the latter will not succeed in eradicating progressing tumor lesions [44]. Based on these models, debulking tumor mass combined with targeted elimination of CD20^+^ cells is assumed to eradicate tumor lesions more rapidly while avoiding the risk of relapse.

To specifically eliminate CD20^+^ melanoma cells, Schmidt and colleagues (2011) [4] made use of genetically engineered cytotoxic T cells, which are redirected by a chimeric antigen receptor recognizing defined tumor cell subsets in a MHC-independent fashion. Alternatively, redirecting T cells by a recombinant T cell receptor in a MHC-dependent manner will be feasible as soon as tumor cells properly present the target antigen. Compared to therapeutic drugs, redirected T cells have the advantage that they actively penetrate tissues and scan cells for specific ligands. Once activated, T cells lyse the cognate cell, secrete a spectrum of pro-inflammatory cytokines and proliferate, which in total results in a forced and prolonged anti-tumor attack. Without contact to the antigen the T cell response ceases, and the majority of T cells undergoes apoptosis. Although some T cells are assumed to persist in the long-term, thus providing CAR-defined antigen-specific memory, definite proof is still lacking.

Adoptive immunotherapy by engineered T cell is still being explored in phase I trials, whereas therapeutic antibodies have been in clinical use for the treatment of malignant diseases for nearly a decade. In contrast to chemotherapy, antibody treatment has the advantage that it is not neutralized by a drug transporter or the dormant stage of the target cell. However, successful treatment of melanomas with anti-CD20 antibodies such as Rituxan rituximab or Arzerra ofatumumab depends on several pre-requisites: sensitivity of CD20^+^ melanoma cells to those antibodies, which is assumed on the basis of B lymphoma cell killing but not yet been demonstrated, efficient penetration into solid tumor tissues to target the few melanoma cells, and therapeutic antibody levels over time, in particular when phenotype shifting plays a major role. When systemically applied, the concomitant elimination of B lymphocytes and the drop in immunoglobulin levels has to be clinically addressed. An explorative phase I melanoma study applying anti-CD20 antibody rituximab as adjuvant for low-dose IL-2 has been reported [45]; however, the study did not determine any benefit in anti-tumor response. Currently, a phase I/II trial using anti-CD20 antibody in the treatment of metastatic melanoma is in planning [46]. As alternative to the therapeutic anti-CD20 antibody, anti-CD20 radio-immunoconjugates including ibritumomab, tiuxetan and tositumomab are also worth testing. Recombinant single-chain antibodies may be advantageous since they penetrate solid tissues more efficiently than full length antibodies. When combined with a T-cell-engaging domain, those recombinant bi-specific antibodies are extremely effective in inducing an anti-tumor cell response in circulation and solid tissues [47].

CSCs in long-term proliferative quiescence (“dormancy”) escape anti-proliferative chemotherapy, which allows tumor relapse even after decades. This was observed in the treatment of CML with imatinib; even complete responses to treatment frequently relapse after discontinuation of treatment. Successful therapy by anti-proliferative drugs therefore has to induce cell cycle entry of CSCs thus making them more sensitive, as recently shown for AML [48]. Moreover, the therapeutic effect of anti-proliferative drugs will be counteracted by the pronounced expression of the chemo-resistance mediator ABCB5 in melanoma CSCs [37]. In general, CSCs are thought to be more resistant to chemotherapy and radiation than the bulk of the tumor cells. This observation is supported by pre-clinical data on CD44^+^CD24^low^ mammary carcinoma CSCs and clinical observations that CSCs remained present and increased in relative numbers in neo-adjuvant chemotherapy, whereas cells without CSC markers regressed [49, 50]. In the context of CD20^+^ melanoma maintaining cells, it will be of particular interest to determine whether the frequency of those cells during chemotherapy correlates with the clinical outcome irrespective of the tumor cell mass. Furthermore, long-term proliferative quiescence of CD20^+^ melanoma cells may counteract clonal evolution of genetic and epigenetic modifications, which continuously occurs and affects different tumor cell subsets in established lesions [51]. Genetic changes may drive resistance to therapy and counteract long-term success; this becomes obvious when mutant BRAF cells change from sensitive to more malignant cells upon treatment with BRAF inhibitors [52]. However, the question of whether or not CD20^+^ melanoma cells undergo substantial clonal evolution during tumor progression needs to be addressed.

If functional and phenotypic plasticity occurs in substantial frequencies in CD20^+^ melanoma cells, the therapeutic targeting of those cells will require an ongoing process and will only be successful in small tumor lesions where the stochastic frequency of newly reverted cells is low. If, after initial treatment, tumor growth relapses in any surviving melanoma cell due to its functional plasticity, eradication of tumor lesions will require the elimination of all melanoma cells and in the long-term will not be possible by targeting of any tumor cell subset at a given time. Conversely, if melanoma growth depends on a fixed tumor cell subpopulation, their specific elimination will effectively eradicate tumor lesions without targeting the bulk of the tumor cells. In their therapeutic approach Schmidt et al. (2011) [4] treated melanoma lesions of about 15-20 mm^3^ in volume, which are fairly well established, have their own stroma and are vascularized, but which represent smaller tumor volumes than human bulk metastases. Moreover, Schmidt and colleagues took advantage of the particular capability of cytolytic T cells to patrol as a guardian through tissues and to eliminate those cells whenever they occur. Targeting the same cells by therapeutic antibodies would require maintenance of therapeutic levels in the long-term; however, this is clinically feasible.

## CONCLUSIONS

Strong evidence for a functional hierarchy in a melanoma lesion is provided by tumor eradication by means of targeted elimination of the minor subset of CD20^+^ melanoma cells. Although isolated individual cancer cells can exhibit “stem-like” capacities under limiting dilution conditions, rare melanoma cells seem to trigger a hierarchy in an established melanoma lesion by maintaining tumor integrity and progression via processes which are currently unknown. Functional and phenotypic plasticity as well as accumulating genetic lesions may result in the situation that the melanoma-maintaining property is not genetically fixed to a certain cell; other master cells which fuel melanoma integrity and progression may exist, in particular in those cases which lack CD20^+^ melanoma cells.

At least three substantial consequences for prognosis and therapy need to be addressed:

First, whether functional plasticity counteracts the therapeutic efficacy of targeted elimination of CD20^+^ melanoma cells in the clinical situation.

Second, whether the frequency of CD20^+^ melanoma cells serves as a surrogate for therapeutic efficacy and is of prognostic value.

Third, whether “dormant” CD20^+^ melanoma cells initiate melanoma relapse even after decades.
